# Supplementary material for: Healthcare costs and outcomes associated with laboratory-confirmed Lyme disease in Ontario, Canada: A population-based cohort study
Source: PLoS One. 2023 Jun 22;18(6):e0286552. doi: 10.1371/journal.pone.0286552 (PMC10286989; doi:10.1371/journal.pone.0286552)
Supplement: S1 File — (DOCX) [file pone.0286552.s001.docx]

S1 File. Case definitions for Lyme disease in Ontario

**Confirmed Case:**

- Clinician-confirmed erythema migrans (EM) greater than five cm in diameter with a history of residence in, or visit to, a Lyme disease endemic area or risk area;

**OR**

- Clinical evidence of Lyme disease with laboratory confirmation by polymerase chain reaction (PCR) or culture;

**OR**

- Clinical evidence of Lyme disease with laboratory support by serological methods, and a history of residence in, or visit to, an endemic area or risk area.

**Probable case:**

- Clinical evidence of Lyme disease with laboratory support by serological methods, with no history of residence in, or visit to an endemic area or risk area;

OR

- Clinician-confirmed erythema migrans (EM) greater than five cm in diameter with no history of residence in, or visit to an endemic area or risk area.

**Source:** Ministry of Health and Long-Term Care. Infectious Diseases Protocol. Appendix B: Provincial Case Definitions for Diseases of Public Health Significance. Disease: Lyme Disease. Effective: February 2019. Accessed January 03, 2022 from: <https://www.health.gov.on.ca/en/pro/programs/publichealth/oph_standards/docs/lyme_disease_cd.pdf>
