# Supplementary material for: Healthcare costs and outcomes associated with laboratory-confirmed Lyme disease in Ontario, Canada: A population-based cohort study
Source: PLoS One. 2023 Jun 22;18(6):e0286552. doi: 10.1371/journal.pone.0286552 (PMC10286989; doi:10.1371/journal.pone.0286552)
Supplement: S1 Table — (DOCX) [file pone.0286552.s002.docx]

S2 Table. Variables included in propensity score regression and matching

| **Variables** | **Description** | **Criteria** |
| --- | --- | --- |
| Propensity score |  |  |
| Rurality | - Rural is a binary variable (1/0) - StatsCan definition, which is defined by ‘CSIZE=5’ (rural and small town -includes all places in Canada with an urban area population < 10,000, plus rural areas | At index date |
| Neighbourhood income quintiles | - A measure of relative household income, adjusted for household size and cost of living - Individuals are then assigned the income quintile of the DA in which they live - Income quintile 1 contains the lowest incomes; income quintile 5 contains the highest incomes | At index date |
| Comorbidities | - Measured two years prior to the index date - Adjusted for using the John Hopkins ACG® System Aggregated Diagnosis Groups (32 binary variables) | 2 year before index date |
| Public health units | - Patient residence from RPDB (4 numbers) | At index date |
| **Matching** |  |  |
| Age | - Age at index date (continuous) | + 5 years |
| Sex | - Male or female (binary) | Exact |
| Logit of propensity score | - From propensity score regression using variables above | Caliper width of 0.2 SDs of logit of propensity score |

DA, dissemination area ; RPDB, registered persons database; SD, standard deviation
