# Supplementary material for: Healthcare costs and outcomes associated with laboratory-confirmed Lyme disease in Ontario, Canada: A population-based cohort study
Source: PLoS One. 2023 Jun 22;18(6):e0286552. doi: 10.1371/journal.pone.0286552 (PMC10286989; doi:10.1371/journal.pone.0286552)
Supplement: S2 Table — (DOCX) [file pone.0286552.s003.docx]

S3 Table. Cost variable definitions and source of data

| **Database** | **Resource utilization variable and descriptions** |
| --- | --- |
| OHIP | The Ontario Health Insurance Plan (OHIP) contains all OHIP claims data for:   - Outpatient physician visits - Laboratory services - Non-physician services |
| NACRS | The National Ambulatory Care Reporting System (NACRS) contains data for all hospital-based and community-based ambulatory care:   - Day surgery - Outpatient and community-based clinics - Emergency departments |
| DAD | The DAD contains demographic, administrative and clinical data for   - Hospital inpatient discharges - Day surgery interventions |
| CAPE | Capitation costs |
| ODB | Medication use* |
| NRS | Rehabilitation admissions |
| CCRS | Complex continuing care admissions  Long-term care |
| HCD | Home care services |
| OMHRS | Mental health admissions |
| ADP | Assistive devices |

*Only individuals who are 65 years and older or on social assistance are included in this database.

CCRS, Continuing Care Reporting System; DAD, Discharge Abstract Database; HCD, Home Care Database; NACRS, National Ambulatory Care Reporting System; NRS, National Rehabilitation Reporting System; ODB, Ontario Drug Benefit; OHIP, Ontario Health Insurance Plan; OMHRS, Ontario Mental Health Reporting System;
