# Supplementary material for: Healthcare costs and outcomes associated with laboratory-confirmed Lyme disease in Ontario, Canada: A population-based cohort study
Source: PLoS One. 2023 Jun 22;18(6):e0286552. doi: 10.1371/journal.pone.0286552 (PMC10286989; doi:10.1371/journal.pone.0286552)
Supplement: S3 Table — (DOCX) [file pone.0286552.s004.docx]

S4 Table. ICD-10 and OHIP codes to identify Lyme disease and potentially related sequelae

| **Sequelae** | **ICD10** | **OHIP** |
| --- | --- | --- |
| **Lyme disease** | **A69.2** Lyme disease  *Includes: Erythema chronicum migrans due to Borrelia burgdorferi* | **079** – Other viral diseases  **136** – Other infections |
| **Arthritic** | **M00*** Pyogenic arthritis  **M01.2*** (M01.20 – M01.29) Arthritis in Lyme disease (A69.2†)  **M06*** Other rheumatoid arthritis  **M08*** Juvenile arthritis  **M13*** Other arthritis  **M25*** Other joint disorders, not elsewhere classified | **711** - Pyogenic arthritis  **714** - Rheumatoid arthritis  **715** - Osteoarthritis  **716** - Traumatic arthritis |
| **Cardiac** | **B33.2** Viral carditis  **I30.0** Acute nonspecific idiopathic pericarditis  **I30.1** Infective pericarditis  **I30.8** Other forms of acute pericarditis  **I30.9** Acute pericarditis, unspecified  **I32.0** Pericarditis in bacterial diseases classified elsewhere  **I32.1*** Pericarditis in other infectious and parasitic diseases classified elsewhere  **I32.8** Pericarditis in other diseases classified elsewhere  **I33*** Acute and subacute infective endocarditis  **I33.9** Acute endocarditis, unspecified  **I38*** Endocarditis, valve unspecified  **I39*** Endocarditis and heart valve disorders in diseases classified elsewhere  **I40.0** Infective myocarditis  **I40.1** Isolated myocarditis  **I40.8** Other acute myocarditis  **I40.9** Acute myocarditis, unspecified  **I41.0** Myocarditis in bacteria diseases classified elsewhere  **I41.1** Myocarditis in viral diseases classified elsewhere  **I41.2** Myocarditis in other infectious and parasitic diseases classified elsewhere  **I41.8** Myocarditis in other diseases classified elsewhere  **I43.0*** Cardiomyopathy in infectious and parasitic diseases classified elsewhere  **I51.4** Myocarditis, unspecified  **I52.1*** Other heart disorders in other infectious and parasitic diseases classified elsewhere | **412** - Old myocardial infarction, chronic coronary artery disease of arteriosclerotic heart disease, without symptoms  **413** - Acute coronary insufficiency, angina pectoris, acute ischaemic heart disease  **426** - Heart blocks, other conduction disorders  **427** - Paroxysmal tachycardia, atrial or ventricular flutter or fibrillation, cardiac arrest, other arrythmias  **428** - Congestive heart failure  **429** - All other forms of heart disease |
| **Cognitive** |  | **307** - Habit spasms, tics, stuttering, tension headaches, anorexia nervosa, sleep disorders, enuresis  **311** - Depressive or other non-psychotic disorders, not elsewhere classified  **315** - Specified delays in development (e.g., dyslexia, dyslalia, motor retardation)  **319** - Mental retardation |
| **Dermatological (skin rashes)** |  | **695** - Erythema multiforme, erythema nodosum, acne, rosacea, lupus erythematosus, intertrigo  **709** - Other disorders of skin and subcutaneous tissue |
| **Neurologic (facial palsy)** | G51.0 Bell’s Palsy  **G53.1** Multiple cranial nerve palsies in infectious and parasitic diseases classified elsewhere (A00-B99†) | **351** - Bell's palsy, facial nerve disorders  **352** - Disorders of other cranial nerves |
| **Neurologic (meningitis)** | **G01*** Meningitis in bacterial diseases classified elsewhere  *Includes: Meningitis (in): *Lyme disease (A69.2†)* | **036** - Meningococcal infection or meningitis  **320** - Bacterial meningitis  **321** - Meningitis due to other organisms |
| **Neurologic Und(polyneuropathy)** | **G63.0*** Polyneuropathy in infectious and parasitic diseases classified elsewhere  *Includes: Polyneuropathy (in): *Lyme disease (A69.2†)* | **356** - Idiopathic peripheral neuritis |
| **Physical** |  | **780** - Convulsions, ataxia, vertigo, headache, except tension headache and migraine  **781** - Leg cramps, leg pain, muscle pain, joint pain, arthralgia, joint swelling, masses  **785** - Chest pain, tachycardia, syncope, shock, edema, masses  **787** - Anorexia, nausea and vomiting, heartburn, dysphagia, hiccough, hematemesis, jaundice, ascites, abdominal pain, melena, masses  **795** - Chronic Fatigue Syndrome |

† Per ICD-10, this symbol indicates a code that represents the etiology or underlying cause of a disease. A code representing the manifestation of the disease should also be recorded. The dagger code should be sequenced before the manifestation code.

* Per ICD-10, this symbol is used to indicate a code that represents the manifestation of a disease. This code should be paired with a dagger (etiology) code and should follow this in sequence.

ICD-10, International Statistical Classification of Diseases and Related Health Problem, Tenth Revision, Canada; OHIP, Ontario Health Insurance Plan
