# Supplementary material for: Healthcare costs and outcomes associated with laboratory-confirmed Lyme disease in Ontario, Canada: A population-based cohort study
Source: PLoS One. 2023 Jun 22;18(6):e0286552. doi: 10.1371/journal.pone.0286552 (PMC10286989; doi:10.1371/journal.pone.0286552)
Supplement: S5 Table — (DOCX) [file pone.0286552.s006.docx]

S6 Table. Laboratory-confirmed LD cases by age and sex (n=2,808)

|  | **Frequency (%)** | |
| --- | --- | --- |
|  | **Male** | **Female** |
| **Total laboratory-confirmed cases (n=2,808)** | 1,563 (55.7%) | 1,245 (44.3%) |
| **Age at index date, years** |  |  |
| Mean (SD) | 45.48 (20.77) | 47.78 (20.44) |
| Median (IQR) | 50 (29 – 61) | 52 (33 – 63) |
| **Age Groups** |  |  |
| < 10 | 112 (4.0%) | 76 (2.7%) |
| 10 to 19 | 132 (4.7%) | 83 (3.0%) |
| 20 to 29 | 148 (5.3%) | 101 (3.6%) |
| 30 to 39 | 156 (5.6%) | 137 (4.9%) |
| 40 to 49 | 231 (8.2%) | 154 (5.5%) |
| 50 to 59 | 327 (11.7%) | 269 (9.6%) |
| 60 to 69 | 293 (10.4%) | 277 (9.9%) |
| 70 to 79 | 133 (4.7%) | 113 (4.0%) |
| > 80 | 31 (1.1%) | 35 (1.3%) |
| **Index year** |  |  |
| 2006 | 15 (0.5%) | 15 (0.5%) |
| 2007 | 28 (1.0%) | 33 (1.2%) |
| 2008 | 30 (1.0%) | 49 (1.8%) |
| 2009 | 31 (1.1%) | 48 (1.7%) |
| 2010 | 38 (1.4%) | 42 (1.5%) |
| 2011 | 54 (1.9%) | 56 (2.0%) |
| 2012 | 70 (2.5%) | 62 (2.2%) |
| 2013 | 142 (5.1%) | 116 (4.1%) |
| 2014 | 94 (3.4%) | 81 (2.9%) |
| 2015 | 163 (5.8%) | 117 (4.2%) |
| 2016 | 145 (5.2%) | 112 (4.0%) |
| 2017 | 488 (17.4%) | 323 (11.5%) |
| 2018 | 265 (9.4%) | 191 (6.8%) |

IQR, interquartile range; LD, Lyme disease; SD, standard deviation
