# Supplementary material for: Healthcare costs and outcomes associated with laboratory-confirmed Lyme disease in Ontario, Canada: A population-based cohort study
Source: PLoS One. 2023 Jun 22;18(6):e0286552. doi: 10.1371/journal.pone.0286552 (PMC10286989; doi:10.1371/journal.pone.0286552)
Supplement: S6 Table — (DOCX) [file pone.0286552.s007.docx]

S7 Table. Baseline characteristics of matched cohort at predeath

|  | **After matching** | | |
| --- | --- | --- | --- |
| **Variables** | **Exposed (LD)**  **(n=38)** | **Unexposed (no LD) (n=110)** | **Weighted Standardized Differences** |
| **Age** |  |  |  |
| Mean ± SD | 72.21 ± 10.65 | 71.75 ± 10.41 | 0.02 |
| Median (IQR) | 72 (65-78) | 72 (65-77) | 0.02 |
| **Sex** |  |  |  |
| Female | 9 (23.7%) | 27 (24.5%) | 0.00 |
| Male | 29 (76.3%) | 83 (75.5%) | 0.00 |
| **Neighbourhood Income Quintile** |  |  |  |
| 1 (lowest) | < 6 (< 13.2%) | 15 (13.6%) | 0.11 |
| 2 | 9 (23.7%) | 30 (27.3%) | 0.07 |
| 3 | < 6 (< 13.2%) | 13 (11.8%) | 0.03 |
| 4 | 12 (31.6%) | 32 (29.1%) | 0.03 |
| 5 (highest) | 9 (23.7%) | 20 (18.2%) | 0.15 |
| Missing | - | - | - |
| **Rural** |  |  |  |
| No | 23 (60.5%) | 68 (61.8%) | 0.02 |
| Yes | 15 (39.5%) | 42 (38.2%) | 0.02 |
| **Public Health Unit** |  |  |  |
| Durham | < 6 (< 13.2%) | < 6 (< 5.5%) | 0.19 |
| Halton | < 6 (< 13.2%) | < 6 (< 5.5%) | 0.00 |
| Hastings and Prince Edward | < 6 (< 13.2%) | 8 (7.3%) | 0.03 |
| Kent-Chatham | < 6 (< 13.2%) | < 6 (< 5.5%) | 0.06 |
| Kingston-Frontenac-Lennox and Addington | < 6 (< 13.2%) | 14 (12.7%) | 0.01 |
| Leeds-Grenville-Lanark | 8 (21.1%) | 19 (17.3%) | 0.04 |
| Ottawa Carleton | < 6 (< 13.2%) | 15 (13.6%) | 0.08 |
| Peel | < 6 (< 13.2%) | < 6 (< 5.5%) | 0.05 |
| Eastern Ontario | < 6 (< 13.2%) | 6 (5.5%) | 0.14 |
| Simcoe | < 6 (< 13.2%) | < 6 (< 5.5%) | 0.15 |
| Waterloo | < 6 (< 13.2%) | < 6 (< 5.5%) | 0.00 |
| Wellington-Dufferin-Guelph | < 6 (< 13.2%) | 10 (9.1%) | 0.15 |
| Windsor-Essex | < 6 (< 13.2%) | < 6 (< 5.5%) | 0.00 |
| York | < 6 (< 13.2%) | < 6 (< 5.5%) | 0.10 |
| City of Toronto | < 6 (< 13.2%) | 11 (10.0%) | 0.03 |
| **Co-morbidities** |  |  |  |
| Time Limited: Minor |  |  |  |
| No | 25 (65.8%) | 81 (73.6%) | 0.16 |
| Yes | 13 (34.2%) | 29 (26.4%) | 0.16 |
| Time Limited: Minor-Primary Infections |  |  |  |
| No | 17 (44.7%) | 50 (45.5%) | 0.02 |
| Yes | 21 (55.3%) | 60 (54.5%) | 0.02 |
| Time Limited: Major |  |  |  |
| No | 30 (78.9%) | 81 (73.6%) | 0.11 |
| Yes | 8 (21.1%) | 29 (26.4%) | 0.11 |
| Time Limited: Major-Primary Infections |  |  |  |
| No | 25 (65.8%) | 66 (60.0%) | 0.12 |
| Yes | 13 (34.2%) | 44 (40.0%) | 0.12 |
| Allergies |  |  |  |
| No | > 86.8%* | > 94.5%* | 0.05 |
| Yes | NR | NR | 0.05 |
| Asthma |  |  |  |
| No | > 86.8%* | > 94.5%* | 0.05 |
| Yes | NR | NR | 0.05 |
| Likely to Recur: Discrete |  |  |  |
| No | 23 (60.5%) | 73 (66.4%) | 0.14 |
| Yes | 15 (39.5%) | 37 (33.6%) | 0.14 |
| Likely to Recur: Discrete-Infections |  |  |  |
| No | 30 (78.9%) | 95 (86.4%) | 0.21 |
| Yes | 8 (21.1%) | 15 (13.6%) | 0.21 |
| Likely to Recur: Progressive |  |  |  |
| No | > 86.8%* | 98 (89.1%) | 0.00 |
| Yes | NR | 12 (10.9%) | 0.00 |
| Chronic Medical: Stable |  |  |  |
| No | 13 (34.2%) | 29 (26.4%) | 0.17 |
| Yes | 25 (65.8%) | 81 (73.6%) | 0.17 |
| Chronic Medical: Unstable |  |  |  |
| No | 17 (44.7%) | 40 (36.4%) | 0.15 |
| Yes | 21 (55.3%) | 70 (63.6%) | 0.15 |
| Chronic Specialty: Stable-Orthopedic |  |  |  |
| No | > 86.8%* | > 94.5%* | 0.06 |
| Yes | NR | NR | 0.06 |
| Chronic Specialty: Stable-Ear, Nose, Throat |  |  |  |
| No | > 86.8%* | > 94.5%* | 0.00 |
| Yes | NR | NR | 0.00 |
| Chronic Specialty: Stable-Eye |  |  |  |
| No | 31 (81.6%) | 93 (84.5%) | 0.05 |
| Yes | 7 (18.4%) | 17 (15.5%) | 0.05 |
| Chronic Specialty: Unstable-Orthopedic |  |  |  |
| No | > 86.8%* | 103 (93.6%) | 0.04 |
| Yes | NR | 7 (6.4%) | 0.04 |
| Chronic Specialty: Unstable-Eye |  |  |  |
| No | 38 (100.0%) | 110 (100.0%) | 0 |
| Yes | 0 (0%) | 0 (0%) | 0 |
| Dermatologic |  |  |  |
| No | > 86.8%* | 93 (84.5%) | 0.13 |
| Yes | NR | 17 (15.5%) | 0.13 |
| Injuries/Adverse Effects: Minor |  |  |  |
| No | 27 (71.1%) | 77 (70.0%) | 0.03 |
| Yes | 11 (28.9%) | 33 (30.0%) | 0.03 |
| Injuries/Adverse Effects: Major |  |  |  |
| No | 29 (76.3%) | 84 (76.4%) | 0 |
| Yes | 9 (23.7%) | 26 (23.6%) | 0 |
| Psychosocial: Time Limited, Minor |  |  |  |
| No | 28 (73.7%) | 78 (70.9%) | 0.06 |
| Yes | 10 (26.3%) | 32 (29.1%) | 0.06 |
| Psychosocial: Recurrent or Persistent, Stable |  |  |  |
| No | > 86.8%* | 94 (85.5%) | 0.12 |
| Yes | NR | 16 (14.5%) | 0.12 |
| Psychosocial: Recurrent or Persistent, Unstable |  |  |  |
| No | 28 (73.7%) | 85 (77.3%) | 0.10 |
| Yes | 10 (26.3%) | 25 (22.7%) | 0.10 |
| Signs/Symptoms: Minor |  |  |  |
| No | > 86.8%* | 102 (92.7%) | 0.11 |
| Yes | NR | 8 (7.3%) | 0.11 |
| Signs/Symptoms: Uncertain |  |  |  |
| No | 17 (44.7%) | 49 (44.5%) | 0 |
| Yes | 21 (55.3%) | 61 (55.5%) | 0 |
| Signs/Symptoms: Major |  |  |  |
| No | 10 (26.3%) | 28 (25.5%) | 0.04 |
| Yes | 28 (73.7%) | 82 (74.5%) | 0.04 |
| Discretionary |  |  |  |
| No | 21 (55.3%) | 65 (59.1%) | 0.10 |
| Yes | 17 (44.7%) | 45 (40.9%) | 0.10 |
| See and Reassure |  |  |  |
| No | > 86.8%* | 97 (88.2%) | 0.04 |
| Yes | NR | 13 (11.8%) | 0.04 |
| Prevention/Administrative |  |  |  |
| No | > 86.8%* | > 94.5%* | 0.00 |
| Yes | NR | NR | 0.00 |
| Malignancy |  |  |  |
| No | 21 (55.3%) | 59 (53.6%) | 0.02 |
| Yes | 17 (44.7%) | 51 (46.4%) | 0.02 |
| Pregnancy |  |  |  |
| No | 31 (81.6%) | 82 (74.5%) | 0.16 |
| Yes | 7 (18.4%) | 28 (25.5%) | 0.16 |
| Dental |  |  |  |
| No | 38 (100.0%) | 110 (100.0%) | 0 |
| Yes | 0 (0%) | 0 (0%) | 0 |

IQR, interquartile range; LD, Lyme disease; NR, not report due to small cell; SD, standard deviation

* Cannot be exact due to small cells
