# Supplementary material for: Healthcare costs and outcomes associated with laboratory-confirmed Lyme disease in Ontario, Canada: A population-based cohort study
Source: PLoS One. 2023 Jun 22;18(6):e0286552. doi: 10.1371/journal.pone.0286552 (PMC10286989; doi:10.1371/journal.pone.0286552)
Supplement: S7 Table — (DOCX) [file pone.0286552.s008.docx]

S8 Table. Mean phase-of-care costs for matched exposed and unexposed individuals

|  | LD Phase of Care | | | |
| --- | --- | --- | --- | --- |
| **Total costs,**  **Mean (95% CI)** | **Pre-diagnosis**  **(10-days)** | **Acute**  **(30-days)** | **Post-acute**  **(150 days)** | **Continuing**  **(Rest of follow-up*)** |
| **Exposed cohort**  **(n=2,772)** | **$209**  **($181, $238)** | **$1,084**  **($956, $1,212)** | **$1,714**  **($1,499, $1,927)** | **$11,013**  **($9,854, $12,172)** |
| Female (n=1,231) | $225  ($178, $272) | $1,063  ($857, $1,270) | $1,719  ($1,460, $1,980) | $12,497  ($11,100, $13,800) |
| Male (n=1,541) | $197  ($163, $232) | $1,101  ($939, 1,260) | $1,709  ($1,390, $2,030) | $9,827  ($8,050, $11,600) |
| **Unexposed cohort**  **(n=8,217)** | **$96**  **($81, $112)** | **$260**  **($226, $294)** | **$1,339**  **($1,221, $1,457)** | **$13,414**  **($12,622, $14,208)** |
| Female (n=3,657) | $99  ($80, $118) | $274  ($224, $324) | $1,474  ($1,290, $1,660) | $15,315  ($14,100, $16,500) |
| Male  (n=4,560) | $95  ($72, $118) | $248  ($202, $294) | $1,231  ($1,080, $1,380) | $11,891  ($10,800, $13,000) |

*Follow-up time varies; mean (SD) was 4.83 (3.06) years

CI, confidence interval; LD, Lyme disease
